# Health-Related Quality of Life and Health Service Use among Multimorbid Middle-Aged and Older-Aged Adults in China: A Cross-Sectional Study in Shandong Province

**DOI:** 10.3390/ijerph17249261

**Published:** 2020-12-11

**Authors:** Qinfeng Zhao, Jian Wang, Stephen Nicholas, Elizabeth Maitland, Jingjie Sun, Chen Jiao, Lizheng Xu, Anli Leng

**Affiliations:** 1 Centre for Health Management and Policy Research, School of Public Health, Cheeloo College of Medicine, Shandong University, Jinan 250012, China; zhaoqinfeng6@mail.sdu.edu.cn (Q.Z.); 201835804@mail.sdu.edu.cn (C.J.); 2 NHC Key Lab of Health Economics and Policy Research (Shandong University), Jinan 250012, China; 3 Dong Fureng Institute of Economics and Social Development, Wuhan University, No. 54 Dongsi Lishi Hutong, Dongcheng District, Beijing 100010, China; wangjian993@whu.edu.cn; 4 Center for Health Economics and Management, Economics and Management School, Wuhan University, Luojia Hill, Wuhan 430072, China; 5 Australian National Institute of Management and Commerce, 1 Central Avenue Australian Technology Park, Eveleigh, NSW 2015, Australia; stephen.nicholas@newcastle.edu.au; 6 Guangdong Institute for International Strategies, Guangdong University of Foreign Studies, 2 Baiyun North Avenue, Guangzhou 510420, China; 7 School of Economics, Tianjin Normal University, No. 339 Binshui West Avenue, Tianjin 300387, China; 8 School of Management, Tianjin Normal University, No. 339 Binshui West Avenue, Tianjin 300387, China; 9 Newcastle Business School, University of Newcastle, University Drive, Newcastle, NSW 2308, Australia; 10 School of Management, University of Liverpool, Chatham Building, Chatham Street, Liverpool L697ZH, UK; e.maitland@liverpool.ac.uk; 11 Shandong Health Commission Medical Management Service Center, Jinan 250012, China; sunjingjie@shandong.cn; 12 UNSW Medicine, UNSW Sydney, Sydney, NSW 2052, Australia; lizheng.xu@unsw.edu.au; 13 The George Institute for Global Health, Newtown, NSW 2042, Australia; 14 School of Political Science and Public Administration, Institute of Governance, Shandong University, 72 Binhai Rd, Qingdao 266237, China

**Keywords:** China, multimorbidity, middle-aged and old-aged adults

## Abstract

(1) Background: The management of multiple chronic diseases challenges China’s health system, but current research has neglected how multimorbidity is associated with poor health-related quality of life (HRQOL) and high health service demands by middle-aged and older adults. (2) Methods: A cross-sectional study was conducted in Shandong province, China in 2018 across three age groups: Middle-aged (45 to 59 years), young-old (60 to 74 years), and old-old (75 or above years). The information about socio-economic, health-related behaviors, HRQOL, and health service utilization was collected via face-to-face structured questionnaires. The EQ-5D-3L instrument, comprising a health description system and a visual analog scale (VAS), was used to measure participants’ HRQOL, and χ^2^ tests and the one-way ANOVA test were used to analyze differences in socio-demographic factors and HRQOL among the different age groups. Logistic regression models estimated the associations between lifestyle factors, health service utilization, and multimorbidity across age groups. (3) Results: There were 17,867 adults aged 45 or above in our sample, with 9259 (51.82%) female and 65.60% living in rural areas. Compared with the middle-aged adults, the young-old and old-old were more likely to be single and to have a lower level of education and income, with the old-old having lower levels than the young-old (*P* < 0.001). We found that 2465 (13.80%) suffered multimorbidities of whom 75.21% were older persons (aged 60 or above). As age increased, both the mean values of EQ-5D utility and the VAS scale decreased, displaying an inverse trend to the increase in the number of chronic diseases (*P* < 0.05). Ex-smokers and physical check-ups for middle or young-old respondents and overweight/obesity for all participants (*P* < 0.05) were positively correlated with multimorbidity. Drinking within the past month for all participants (*P* < 0.001), and daily tooth-brushing for middle (*P* < 0.05) and young-old participants (*P* < 0.001), were negatively associated with multimorbidity. Multimorbidities increased service utilization including outpatient and inpatient visits and taking self-medicine; and the probability of health utilization was the lowest for the old-old multimorbid patients (*P* < 0.001). (4) Conclusions: The prevalence and decline in HRQOL of multimorbid middle-aged and older-aged people were severe in Shandong province. Old patients also faced limited access to health services. We recommend early prevention and intervention to address the prevalence of middle-aged and old-aged multimorbidity. Further, the government should set-up special treatment channels for multiple chronic disease sufferers, improve medical insurance policies for the older-aged groups, and set-up multiple chronic disease insurance to effectively alleviate the costs of medical utilization caused by economic pressure for outpatients and inpatients with chronic diseases.

## 1. Introduction

Chronic diseases challenge public health systems and individual well-being around the world. In China, chronic diseases accounted for 89% of all deaths and 70% of disability-adjusted life-years lost, with devastating impacts on individuals, the public health system, and the social economy [1,2,3,4]. With population ageing, and lifestyle changes driving health risk factors, the prevalence of multimorbidity, or two or more chronic conditions, is intensifying, and multimorbidity is a major concern for chronic diseases management [5]. Multimorbidity prevalence around the world varies by age, ranging from 2.6% to over 95%, and data from China estimate that multimorbidity ranges from 6.4% to 76.5% among people aged over 60 years old [1,6]. Multimorbidity leads to higher mortality [7] and poorer quality of life, including constraining daily activities, discomfort and pain, decreasing happiness, and the loss of independence [8,9]. Multimorbidity also increases the frequency of hospitalization, outpatient visits, and use of medicine, and imposes health-related financial stress, including catastrophic health expenditure by individuals and families [10,11,12]. Overall, whether for individuals, households, the health system, or society, the multimorbidity presents severe challenges.

Numerous studies have explored the age, gender, place of residence, education, income level, and occupation as factors associated with multimorbidity [12,13,14]. Lifestyle factors, such as obesity, tobacco use, drinking and physical inactivity, and unhealthy diets, have also been identified as multimorbidity risk factors [1,15,16,17,18]. Most of these studies have focused on older adults, given the susceptibility of older people to multimorbidity [10,15,19,20], with global multimorbidity rates as high as 98% among older adults [15]. Current research has tended to neglect how multimorbidity is significantly associated with a poor health-related quality of life (HRQOL) and high health service demands by older adults [9,21]. However, multimorbidity is not only an old person problem. The prevalence of multimorbidity across working-age adults is widespread, impacting the HRQOL of middle-aged adults and imposing high health service demands on the health system [10,22]. For example, the World Health Organization (WHO) estimated that a quarter of chronic disease deaths occur among adults under 45 years old [8]. A Scottish study indicated that 30% of people aged 45 to 64 years suffer from more than one chronic disease [5], and a survey of the general Alberta Canada population reported that 70% of people with multimorbidity were less than 65 years old [6].

To address these lacunae, we assess multimorbidity among both middle-aged and old-aged people in China. Considering the increase in the average life span, we further subdivided the older group into young-old (60 years to 74 years old) and old-old (75 years old and above) according to the WHO age criteria. Using 2018 cross-sectional data from Shandong province, we estimate the health status of multimorbid middle-aged, young-old, and old-old. Second, we retrospectively explored the association between physical multimorbidity and lifestyle factors to discover the reasons for multimorbidity. Third, we explored health-care service use by multimorbid middle-aged and old-aged persons in Shandong.

## 2. Materials and Methods

### 2.1. Data and Sample

Located in eastern China, Shandong province is the second-most populated province and one of China’s rapidly developing industrial provinces [23]. Our data came from the Sixth Health Service Survey of Shandong, which was a part of the 2018 Chinese National Health Service Survey [24]. The Sixth Health Service Survey was conducted by the Health Committee of Shandong Province using a three-stage stratified cluster sampling method. First, 20 counties were randomly selected from 137 counties throughout eastern, central, and western areas in Shandong province as the primary sampling units (PSUs). Second, five townships were randomly selected from each PSU yielding 100 townships comprising 50 urban and 50 rural secondary sampling units (SSUs). Third, two villages were randomly selected from each SSU, providing 200 villages in total. After informed consent, face-to-face interviews using a structured questionnaire were conducted with all members in every selected household, with 35,264 individuals in 12,938 households completing the survey. The inclusion criteria in our study were respondents aged over 45 years old, without cognitive impairments, and we excluded some questionnaires that were uncompleted. In the end, a total of 17,867 subjects were eligible for our study.

### 2.2. Socio-Demographic Variables

Socio-demographic variables included age groups (45–59, 60–74, 75+), sex, marital status (single, married, or partnered), education (no education, primary school, secondary school, high school, and above), residence (rural-urban), and income quintiles level (quintile 1: RMB0–10,000; quintile 2: RMB10,001–20,000; quintile 3: RMB20,001–40,000; quintile 4: RMB40,001–60,000; quintile 5: RMB60,001–1,000,000).

### 2.3. Multimorbidity

Multimorbidity-related conditions were solicited by a range of self-answered disease questions: “Have you been diagnosed with hypertension or diabetes?” “Have you been diagnosed with any chronic diseases except hypertension and diabetes?” and “What chronic diseases do you suffer from?” Each answer was scientifically assessed by investigators separately. Respondents with two or more diseases were classified as multimorbid.

### 2.4. HRQOL

Tested for China, the EQ-5D-3L instrument was used to measure participants’ HRQOL. EQ-5D-3L consists of a health description system and a visual analog scale (VAS). The health description system has five dimensions (“Mobility,” “Self-Care,” “Usual Activities,” “Pain/Discomfort,” and “Anxiety/Depression”), with each dimension described by a single item divided into three levels: No problem, moderate problems, and severe problems. We described 243 health states, where each state was assigned a utility weight between −0.149 and 1, using a utility scoring function derived from the Chinese general population [25]. VAS recorded the respondents’ overall health status ranging from 0 to 100, with higher VAS values representing higher HRQOL [25].

### 2.5. Lifestyle Characteristics

Lifestyle characteristics were assessed by the following responses: “Never-smoker, ex-smoker, current smoker”; “drank within last 30 days, drank more than 30 days ago, never-drank”; “exercise times ≤1 or ≥2 weekly”; The classifications of BMI were underweight (BMI<18.50 kg/m^2^), normal weight (BMI 18.50–24.99 kg/m^2^), overweight (BMI 25.00–29.99 kg/m^2^), and obese (BMI ≥ 30.00 kg/m^2^); physical examination in the past year (yes-no); and brushing teeth daily (yes-no).

### 2.6. Health Service Utilization

Health service utilization was assessed through yes-no responses to the following questions: “Have you visited the doctors because of discomfort within two weeks?”; “Have you taken self-medicine because of discomfort within the past two weeks?”; “Have you hospitalized because of discomfort within the past year?”

### 2.7. Statistical Analysis

Participants were divided into three categories: The middle-aged (45 to 59 years), young-old (60 to 74 years), and old-old (75 and above years). The basic variables were described using counts (percentages) and tested using chi-square tests. One-way ANOVA tests were used to compare differences in HRQOL between different numbers of chronic diseases among the three age groups. The associations between lifestyle factors, health service utilization, and multimorbidity across different age groups were estimated by a logistic regression model controlling for socio-economic variables including sex, urban-rural residence, marital status, education, and income levels. The outcomes were expressed as odds ratios (ORs) and 95% confidence intervals (CIs), where *P* < 0.05 denoted statistically significant differences. All data were analyzed using STATA 14.0.

## 3. Results

### 3.1. Respondents’ Characteristics

Table 1 presents the characteristics of the 17,867 respondents stratified by age group, with 49.63% aged 45 to 59 years, 40.58% aged 60 to 74 years, and 9.79% aged over 75 years; 9259 (51.82%) were female and 65.60% participants lived in rural areas. The number of single respondents increased with age (*P* < 0.001). Compared with the middle-aged adults, the young-old and the old-old had a lower level of education and income, with the old-old having lower education and income than the young-old (*P* < 0.001). About 70% of respondents never smoked or drank, and few people chose to quit smoking; and middle-aged participants were more likely to be smokers (23.12%) and drinkers (29.60%) than the young-old (smokers 20.43% and drinkers 20.90%) and the old-old (smokers 16.17% and drinkers 18.51%) (*P* < 0.001). About half (52.39%) of young-old exercised twice or more per week compared to 45.79% of middle-aged and 48.46 of old-old respondents, (*P* < 0.001). In terms of BMI, 24.23% of the old-old were overweight and 4.06% obese, while 38.19% of the middle-aged were overweight and 6.64% obese (*P* < 0.001). About 79% of older people completed health check-ups in the past year, which was significantly higher than the middle-aged (36%) (*P* < 0.001). The proportion of respondents brushing their teeth every day decreased with age (*P* < 0.001).

### 3.2. Prevalence of Multimorbidity

**Figure 1:**
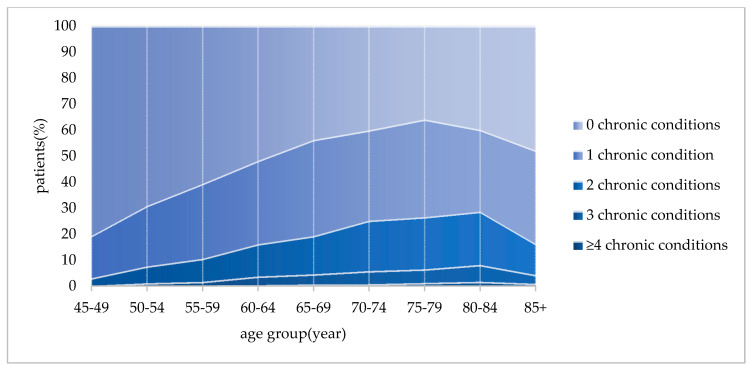
Prevalence of multimorbidity by different age groups.

As shown in Figure 1, the average occurrence of multimorbidity was 13.80% for all 17,867 participants; 6.89% of those aged 45 to 59 years; 19.43% of young-old; and 25.43% of old-old respondents. Further, 70.73% of middle-aged adults had no chronic diseases, and 22.38% suffered from one chronic disease condition. Figure 1 shows that the percent of people with chronic diseases increased with age until 80 years old when it decreased. Among all age groups, the occurrence of multimorbidity was highest (28.71%) in the 80 to 85 aged group.

### 3.3. HRQOL of Respondents with Multimorbidity

Table 2 reveals statistically significant differences in both mean EQ-5D utility values and VAS scores between different numbers of noncommunicable diseases (NCDs) categories (*P* < 0.001) by the three age groups. As age increased, both the mean EQ-5D utility values and VAS scores decreased, and the trend to higher numbers of NCDs categories saw lower EQ-5D utility values and VAS scores. The difference between EQ-5D utility values of multimorbid patients (0.84 ± 0.19) and the mean utility value of total persons (0.92 ± 0.10) in the middle age group was the largest in all age groups, and the VAS scores were also like this in Table 2.

### 3.4. Lifestyle Factors Associated with Multimorbidity

Table 3 shows the results of the logistic regression models to separately identify the association of lifestyle factors with multimorbidity for each age group after controlling for residence, sex, marital status, education, and income level variables. The middle-aged (OR 2.62, 95%CI 1.77 to 3.87) and young-old (OR 1.49, 95%CI 1.17 to 1.89) respondents who were ex-smokers had a higher likelihood of multimorbidity than nonsmokers. People who drank alcohol within the last 30 days in the middle-aged (OR 0.57, 95%CI 0.44 to 0.73), young-old (OR 0.57 95%CI 0.47 to 0.68), and old-old (OR 0.52, 95%CI 0.36 to 0.75) groups had a negative association with multimorbidity compared to nondrinkers. When BMI exceeded the recommended healthy standard, participants were prone to multimorbidity (*P* < 0.01), and being underweight protected old-old respondents from multimorbidity (OR 0.61, 95%CI 0.41 to 0.93). Tooth-brushing had a negative association with the prevalence of multimorbidity among the middle-aged group (OR 0.67, 95%CI 0.52 to 0.88) and young-old group (OR 0.79, 95%CI 0.68 to 0.91). A physical check-up in the past year had a positive impact on the occurrence of multimorbidity among middle-aged (OR 1.64, 95%CI 1.38 to 1.96) and the young-old (OR 1.64, 95%CI 1.43 to 1.89) participants.

### 3.5. Association of Health Service Utilization with Multimorbidity

Table 4 presents the logistic regression model coefficient for the association between outpatient visits, inpatient hospital visits, taking self-medicine, and multimorbidity across the three age groups. There were significant differences in outpatient and inpatient visits and taking self-medicine between multimorbid and nonmultimorbid participants, with multimorbidity increasing the likelihood of service utilization compared with people without multimorbidities (*P* < 0.001). Table 4 also shows that multimorbid younger participants had a higher probability of health service utilization than multimorbid older respondents. The odds ratio of taking self-medicine was higher than that of outpatient visits and inpatient visits. With regard to middle-aged participants, the odds ratio of taking self-medicine was 9.78, which was more than 2.8 times higher than the odds ratio of outpatient visits and 2.09 times higher than the odds ratio of inpatient visits. Income level was also negatively correlated with the utilization of health services in general, and the higher income group had less healthcare utilization.

## 4. Discussion

To our knowledge, this is the first study to examine multimorbidity, HRQOL, health-related behaviors, and health service use across three age groups: Middle-aged adults (45 to 59 years), young-old adults (60 to 74 years), old-old adults (75 and above years) in China. We found that the overall prevalence of multimorbidity was 13.80% for all participants, 6.89% for middle-aged participants, and 20.60% for old-aged participants aged 60 years and above. Multimorbidity among Shandong older-aged persons (20.60%) was higher than rural older-aged people in Yunnan province in 2017 (16.10%) [2], but relatively low compared to another Shandong study in 2017 (35.20%) [20] and a China-wide study (from 6.4 to 76.5%) [1]. These variations in prevalence of older-aged multimorbidity was due to different data sources, sample sizes, differences in type of chronic conditions, different age compositions, and varied definitions of multimorbidity. We found that the likelihood of multimorbidity increased with age, which is consistent with studies from Finland, Poland, Spain, China, Ghana, India, Mexico, and Russia [26]. However, multimorbid prevalence culminated in the 80 to 84 years old group and then fell in our study. Those over 85 years old may be underrepresented in our sample and those without multimorbidity were relatively healthy and lived longer.

The highest mean value of EQ-5D utility and VAS was achieved by middle-aged participants without chronic diseases. HRQOL declined with increasing multimorbidity and age, which is consistent with other studies [27,28,29,30]. However, multimorbidity in middle-aged respondents saw their HRQOL fall relative to the older-aged group. While the older-aged were the most vulnerable to multimorbidity, especially the old-old group and those 80–84 years old, multimorbidity impacted middle-aged participants’ HRQOL more significantly than the older-aged group. This suggests the need to pay more attention not only to the physical, but also the psychological, health of multimorbid middle-aged persons, including actively providing psychological counseling and rehabilitation.

After controlling for basic socio-economic factors, our study found that various lifestyle factors were associated with the prevalence of multimorbidity. Ex-smoker status, obesity, and overweight were positively associated with the probability of multimorbidity, which aligns with previous reports [15,20,31,32,33]. Physical check-ups were more likely to unearth multimorbidity in middle-aged and young-old participants, which suggests that younger people should take regular physical examinations. As demonstrated in numerous studies [16,17,18], exercise was a protective factor to avoid multimorbidity, but the effect was only significant for those over aged 75 in our study. A new finding is that daily teeth brushing could effectively attenuate multimorbidity for middle aged and young-old participants. As periodontal diseases have been proved to be risk factors for cardiovascular diseases, diabetes, rheumatoid arthritis, cancer, and chronic obstructive pulmonary disease [34], it is necessary to develop correct oral cleaning habits from childhood. Drinking within the last 30 days had a negative association with the occurrence of multimorbidity, which contrasts with other studies that reported both current drinking and ex-drinking status were important multimorbid risk factors [15]. Future studies should collect more detailed information about drinking status, such as frequency and amount. Shandong has a unique drinking custom that was different from other Chinese provinces [35], so future studies should collect data on drinking habits in other provinces.

Multimorbidity was positively associated with health service utilization including outpatient and inpatient hospital visits, and taking self-medicine. This aligns with the previous studies conducted in China and high-income countries, which showed that multimorbidity was positively associated with substantially increased use of health care [36,37,38,39]. We also found that health service utilization decreased with the age of participants with multimorbidity, due to older respondents having more limited access to health care, including outpatient and inpatient hospital visits and a greater reliance on self-medicine, compared to younger respondents. In China, older-aged persons tended to have more limited economic capacity and lived away from health facilities. We recommend that the government provide better health service access for the older population, especially older people with a chronic disease or multimorbidity; improve access to health services generally; and provide high-quality health care in order to serve vulnerable people.

There were two major limitations to our study. First, our cross-sectional design meant that causality could not be inferred. Second, the prevalence of chronic disease could be under-reported because we relied on self-reported disease status by subjects who may not be fully aware of their health status. Despite these limitations, our data provided evidence on HRQOL and health service utilization from a comprehensive survey on multimorbidity among the middle-aged and older-aged population in the second-most populated province in China.

## 5. Conclusions

The prevalence and decline in HRQOL of multimorbid middle-aged and older-aged people were severe in Shandong province. Although older-aged people were more susceptible to multimorbidity, multimorbidity caused middle-aged respondents to experience a lower HRQOL. The association between lifestyle factors and multimorbidity was different among the middle-aged, young-old, and old-old participants. We recommend early prevention and intervention to address the prevalence of middle-aged and older-aged multimorbidity, which requires government to improve the diagnostics of chronic diseases, management of multimorbidity, prevention of chronic disease, and health literacy from a young age. Older-aged participants also had limited access to health services. Government should pay close attention to older-aged patients’ access to health services, set-up special treatment channels for sufferers from multiple chronic diseases, improve medical insurance policies for older-aged groups, and set-up multiple chronic disease insurances to effectively alleviate the costs of medical utilization caused by economic pressure for outpatients and inpatients with chronic diseases.

## Figures and Tables

**Table 1:** Descriptive characteristics of study samples by age groups (n, %).

	Total	Middle	Young-Old	Old-Old	
Characteristic n (%)	17867	8867 (49.63)	7250 (40.58)	1750 (9.79)	*P* Value
Sex					0.850
Male	8608 (48.18)	4253 (47.96)	3509 (48.40)	846 (48.34)	
Female	9259 (51.82)	4614 (52.04)	3741(51.60)	904 (51.66)	
Residence					0.038
Rural	11,720 (65.60)	5897 (66.51)	4686 (64.63)	1137 (64.97)	
Urban	6147 (34.40)	2970 (33.49)	2564 (35.37)	613 (35.03)	
Marital status					<0.001
Single	1770 (9.91)	318 (3.59)	791 (10.91)	661 (37.77)	
Couple	16,097 (90.09)	8549 (96.41)	6459 (89.09)	1089 (62.23)	
Education					<0.001
No education	3484 (19.50)	695 (7.84)	2010 (27.72)	779 (44.51)	
Primary school	4746 (26.56)	1848 (20.84)	2323 (32.04)	575 (32.86)	
Secondary school	6268 (35.08)	4168 (47.01)	1865 (25.72)	235 (13.43)	
High school and above	3369 (18.86)	2156 (24.31)	1052 (14.51)	161 (9.20)	
Income quintiles					<0.001
RMB0–10,000	4445 (24.88)	1057(11.92)	2517(34.72)	871(49.77)	
RMB10,001–20,000	2898 (16.22)	1339 (15.10)	1384 (19.09)	175 (10.00)	
RMB20,001–40,000	4475 (25.05)	2863 (32.29)	1358 (18.73)	254 (14.51)	
RMB40,001–80,000	3045 (17.04)	1900 (21.43)	931 (12.84)	214 (12.23)	
RMB80,001–1,000,000	3004 (16.81)	1708 (19.26)	1060 (14.62)	236 (13.49)	
Smoking					<0.001
Never-smoker	13,110 (73.38)	6562 (74.00)	5231 (72.15)	1317 (75.26)	
Ex-smoker	943 (5.28)	255 (2.88)	538 (7.42)	150 (8.57)	
Current-smoker	3814 (21.35)	2050 (23.12)	1481 (20.43)	283 (16.17)	
Drinking					<0.001
Never-drink	12,290 (68.79)	5882 (66.34)	5076 (70.01)	1332 (76.12)	
Drank more than 30 days ago	823 (4.61)	360 (4.06)	369 (5.09)	94 (5.37)	
Drank within last 30 days	4754 (26.61)	2625 (29.60)	1805 (24.90)	324(18.51)	
Number of weekly exercise times					<0.001
<=1	9161 (51.27)	4807 (54.21)	3452 (47.61)	902 (51.54)	
>=2	8706 (48.73)	4060 (45.79)	3798 (52.39)	848 (48.46)	
BMI					<0.001
Underweight	664 (3.72)	138 (1.56)	313 (4.32)	213 (12.17)	
Normal	9874 (55.26)	4754 (53.61)	4078 (56.25)	1042 (59.54)	
Overweight	6268 (35.08)	3386 (38.19)	2458 (33.90)	424 (24.23)	
Obese	1061 (5.94)	589 (6.64)	401 (5.53)	71 (4.06)	
Physical check-up last year					<0.001
No	7539 (42.20)	5005 (56.45)	2167 (29.89)	367 (20.97)	
Yes	10,328 (57.80)	3862 (43.55)	5083 (70.11)	1383 (79.03)	
Teeth-brushing daily					<0.001
No	3578 (20.03)	834 (9.41)	1928 (26.59)	816 (46.63)	
Yes	14,289 (79.97)	8033 (90.59)	5322 (73.41)	934 (53.37)	

**Table 2:** The health-related quality of life (HRQOL) of persons with multimorbidity by age groups (mean ± SD).

Age Groups		Middle		Young-Old		Old-Old	
		EQ-5D Utility Values	VAS Scores	EQ-5D Utility Values	VAS Scores	EQ-5D Utility Values	VAS Scores
	Mean	0.92 ± 0.10	80.80 ± 16.27	0.88 ± 0.15	73.25 ± 17.81	0.78 ± 0.23	66.57 ± 19.00
Number of Noncommunicable diseases (NCDs)	0	0.94 ± 0.07	84.38 ± 13.75	0.91 ± 0.11	78.55 ± 15.69	0.83 ± 0.21	71.07 ± 18.26
	1	0.90 ± 0.12	74.50 ± 17.55	0.87 ± 0.15	71.39 ± 17.35	0.78 ± 0.22	66.72 ± 18.00
	>=2	0.84 ± 0.19	64.49 ± 19.36	0.81 ± 0.21	63.96 ± 18.85	0.71 ± 0.26	59.45 ± 19.36
*P* value		<0.001	<0.001	<0.001	<0.001	<0.001	<0.001

**Table 3:** The association of lifestyle factors with multimorbidity by age groups (odds ratio (OR), 95% confidence interval (CI)).

	Multimorbidity (Reference: No)		
	Middle	Young-Old	Old-Old
Urban (reference: Rural)	1.15	1.24 *	0.98
	(0.94 to 1.40)	(1.08 to 1.44)	(0.73 to 1.30)
Male (reference: Female)	1.28 *	1.12	0.98
	(1.00 to 1.62)	(0.95 to 1.32)	(0.74 to 1.30)
Couple (reference: Single)	1.16	0.91	0.96
	(0.75 to 1.79)	(0.75 to 1.10)	(0.76 to 1.23)
Education (reference: No education)			
Primary school	0.63 *	1.02	0.91
	(0.47 to 0.85)	(0.87 to 1.19)	(0.68 to 1.20)
Secondary school	0.51 ***	0.90	0.82
	(0.38 to 0.68)	(0.75 to 1.09)	(0.56 to 1.23)
High school and above	0.58 ***	0.97	1.62 *
	(0.42 to 0.81)	(0.78 to 1.22)	(1.02 to 2.55)
Income quintiles (reference: RMB0–10,000)			
RMB10,001–20,000	0.80	0.73 ***	1.24
	(0.60 to 1.06)	(0.61 to 0.88)	(0.85 to 1.83)
RMB20,001–40,000	0.65 ***	0.87	1.22
	(0.50 to 0.83)	(0.72 to 1.04)	(0.86 to 1.72)
RMB40,001–60,000	0.52 ***	0.82	1.19
	(0.39 to 0.69)	(0.66 to 1.01)	(0.81 to 1.76)
RMB60,001–1,000,000	0.41 ***	1.29 *	1.31
	(0.30 to 0.56)	(1.05 to 1.58)	(0.87 to 1.97)
Smoking (reference: never-smoker)			
Ex-smoker	2.62 ***	1.49 ***	1.29
	(1.77 to 3.87)	(1.17 to 1.89)	(0.84 to 1.97)
Current-smoker	1.06	0.76 *	0.69
	(0.81 to 1.38)	(0.62 to 0.93)	(0.46 to 1.02)
Drinking (reference: never-drinker)			
Drank more than 30 days ago	1.02	1.14	1.04
	(0.69 to 1.51)	(0.87 to 1.50)	(0.61 to 1.76)
Drank within last 30 days	0.57 ***	0.57 ***	0.52 ***
	(0.44 to 0.73)	(0.47 to 0.68)	(0.36 to 0.75)
BMI (reference: Normal)			
Underweight	1.78	0.99	0.61 *
	(0.99 to 3.19)	(0.72 to 1.36)	(0.41 to 0.93)
Overweight	1.77 ***	1.67 ***	1.98 ***
	(1.48 to 2.12)	(1.47 to 1.90)	(1.53 to 2.56)
Obese	2.43 ***	2.35 ***	2.29 ***
	(1.82 to 3.24)	(1.85 to 2.97)	(1.38 to 3.80)
Number of weekly exercise times (reference: ≤1)			
≥2	1.22 *	0.93	0.72 *
	(1.02 to 1.46)	(0.82 to 1.05)	(0.57 to 0.92)
Tooth-brushing (reference: no)			
Yes	0.67 *	0.79 ***	1.12
	(0.52 to 0.88)	(0.68 to 0.91)	(0.87 to 1.45)
Physical check-up last year (reference: no)			
Yes	1.64 ***	1.64 ***	1.06
	(1.38 to 1.96)	(1.43 to 1.89)	(0.80 to 1.41)

* *P* < 0.05, *** *P* < 0.01.

**Table 4:** Health service utilization of multimorbidity by age groups (OR, 95%CI).

Characteristic	Outpatient (Reference: no)			Self-Medicine (Reference: no)			Inpatient (Reference: no)		
	Middle	Young-Old	Old-Old	Middle	Young-Old	Old-Old	Middle	Young-Old	Old-Old
Multimorbidity	3.49 ***	2.89 ***	2.88 ***	9.78 ***	6.05 ***	3.96 ***	4.66 ***	2.96 ***	2.50 ***
(reference: No)	(2.92 to 4.18)	(2.54 to 3.29)	(2.26 to 3.69)	(8.17 to 11.71)	(5.32 to 6.89)	(3.13 to 5.00)	(3.79 to 5.73)	(2.57 to 3.42)	(1.95 to 3.20)
Residence	0.78 ***	0.96	0.80	1.20 *	1.30 ***	1.25	0.97	0.91	1.11
(reference: Rural)	(0.67 to 0.90)	(0.83 to 1.10)	(0.59 to 1.08)	(1.06 to 1.36)	(1.16 to 1.47)	(0.98 to 1.61)	(0.80 to 1.17)	(0.77 to 1.07)	(0.83 to 1.49)
Gender	0.90	0.94	1.01	0.93	0.80 ***	0.87	1.01	1.07	1.54 ***
(reference: Female)	(0.79 to 1.02)	(0.83 to 1.07)	(0.77 to 1.31)	(0.83 to 1.04)	(0.71 to 0.89)	(0.70 to 1.09)	(0.85 to 1.19)	(0.92 to 1.23)	(1.18 to 2.02)
Marital status	0.92	0.80 *	0.88	1.14	0.94	1.11	1.17	1.40 *	1.10
(reference: Single)	(0.68 to 1.25)	(0.67 to 0.95)	(0.68 to 1.13)	(0.84 to 1.53)	(0.80 to 1.11)	(0.89 to 1.38)	(0.75 to 1.82)	(1.11 to 1.76)	(0.85 to 1.43)
Education (reference: No education)									
Primary school	0.82	0.84 *	0.87	1.01	1.24 *	1.16	1.07	0.97	0.77
	(0.66 to 1.03)	(0.72 to 0.98)	(0.65 to 1.16)	(0.80 to 1.27)	(1.07 to 1.42)	(0.90 to 1.48)	(0.78 to 1.47)	(0.81 to 1.16)	(0.58 to 1.04)
Secondary school	0.67 ***	0.81 *	0.84	0.99	1.41 ***	0.99	1.00	0.95	0.69
	(0.54 to 0.82)	(0.68 to 0.96)	(0.56 to 1.27)	(0.80 to 1.23)	(1.20 to 1.64)	(0.71 to 1.39)	(0.74 to 1.36)	(0.77 to 1.16)	(0.46 to 1.04)
High school and above	0.58 ***	0.86	1.16	1.06	1.13	1.26	0.99	0.82	0.75
	(0.46 to 0.75)	(0.69 to 1.07)	(0.71 to 1.88)	(0.83 to 1.34)	(0.93 to 1.36)	(0.82 to 1.93)	(0.70 to 1.40)	(0.64 to 1.06)	(0.46 to 1.22)
Income (reference: RMB0–10,000)									
RMB10,001–20,000	0.94	0.73 ***	0.59 *	0.87	1.02	1.01	0.95	0.86	1.05
	(0.76 to 1.16)	(0.61 to 0.86)	(0.38 to 0.92)	(0.71 to 1.06)	(0.88 to 1.19)	(0.71 to 1.43)	(0.72 to 1.26)	(0.70 to 1.04)	(0.69 to 1.58)
RMB20,001–40,000	0.89	0.70 ***	0.98	0.77 *	0.98	0.90	0.86	0.92	1.15
	(0.74 to 1.08)	(0.59 to 0.83)	(0.69 to 1.40)	(0.64 to 0.92)	(0.84 to 1.14)	(0.66 to 1.23)	(0.66 to 1.10)	(0.75 to 1.12)	(0.80 to 1.65)
RMB4000–60,000	0.72 *	0.68 ***	0.71	0.88	0.94	1.69 *	0.71 *	0.95	1.11
	(0.58 to 0.89)	(0.55 to 0.84)	(0.47 to 1.09)	(0.73 to 1.07)	(0.79 to 1.13)	(1.19 to 2.38)	(0.53 to 0.95)	(0.75 to 1.20)	(0.74 to 1.67)
RMB60,001–1,000,000	0.69 *	0.77 *	0.95	0.76 *	1.13	1.16	0.71 *	1.05	1.12
	(0.55 to 0.87)	(0.63 to 0.95)	(0.62 to 1.47)	(0.62 to 0.94)	(0.94 to 1.35)	(0.80 to 1.68)	(0.53 to 0.97)	(0.83 to 1.32)	(0.72 to 1.72)

* *P* < 0.05, *** *P* < 0.001.
